# Cephalometric assessment of the hyoid bone position in Oral Breathing Children

**DOI:** 10.1016/S1808-8694(15)31121-6

**Published:** 2015-10-20

**Authors:** Maria Julia Pereira Coelho Ferraz, Darcy Flávio Nouer, José Ricardo Teixeira, Fausto Bérzin

**Affiliations:** 1 M.S. In Orthodontics. Member of CEBAPE - Biosciences Center Applied to Patients with Special Needs / UNESP. PhD Student in Buccodental Biology - anatomy department - FOP/UNICAMP, DDS; 2 PhD. Professor of Orthodontics - Faculdade de Odontologia de Piracicaba/ UNICAMP and Head of the Department of Pediatric Dentistry - Faculdade de Odontologia de Piracicaba/ UNICAMP; 3 MD. Otorhinolaryngologist - AMB. Preceptor at the ENT Medical Residency -Núcleo de ORL de Limeira (NOL); 4 PhD. Full Professor at the Department of Morphology - FOP/UNICAMP

**Keywords:** cephalometry, hyoid bone, oral breathing

## Abstract

**Summary:**

Material and Methods: because of its anatomical and functional relationship with the craniofacial complex, we assessed the cephalometry of the hyoid bone position in relation to the respiratory pattern of these 53 female children, with average age of 10 years; 28 of them are nasal breathers and 25 are oral breathers. Horizontal, vertical and angular cephalometric measures were used in order to determine the hyoid bone location. The Student “t” and the Pearson correlation tests were used in order to compare the groups and the variables.

**Results:**

We did not see statistically significant differences in mandible and hyoid bone positions and the respiratory pattern. In the hyoid triangle, the 0.40 correlation coeficient was significant between AA-ENP (distance between the Atlas vertebrae and the posterior nasal spine) and C3-H (distance between the third cervical vertebrae and the hyoid bone) showing a positive relation between the bony limits of the upper and lower air spaces. For cranial measures we have suggested a relationship between the hyoid bone position and the mandible morphology.

**Conclusion:**

The results led us o conclude that the hyoid bone keeps a stable position, probably in order to secure correct ratios in the airways, and it does not depend on the respiratory pattern.

## INTRODUCTION

Based on the complexity of the stomatognathic system, specific knowledge on its anatomy, physiology and craniofacial growth theories are paramount in order to understand its whole functioning in individuals.

According to Meredith^1^, an important growth increment happens in the first years of life. At birth, the craniofacial bones of a caucasian American corresponds to 60% of the adult head size, 80% at six months of age, 90% at three years, and 95% at nine years of age. Thus, at 12 years of age, when many orthodontists start treatment, almost all facial growth is completed.

Regarding oral breathing, it is possible to pin it as a cause of malocclusion and related areas. Inadequate breathing patterns cause functional adaptations, promoting facial muscle balance, postural changes such as open lips, posterior head tilt and a lower position for mandible and tongue. Consequent to such unbalances, there may be undesirable changes to the craniofacial morphology. Nonetheless, data behind these statements are limited and obscure^2^, ^3^, ^4^, ^5^, ^6^, ^7^.

The importance of the hyoid bone is related to its single relation, nonetheless, it provides connections to pharynx, mandible and cranial muscles, ligaments and fascia.^8^,^9^.

Many of the characteristics of the so called Long Face Syndrome (LFS) group and the Short Face Syndrome (SFS) group could be explained based on the clockwise and counter-clock wise rotation of the mandible “in harmony” with the hyoid bone, tongue, pharynx and neck. The vital need to keep the air space pattern at the tongue base may explain this rotation in the LFS^10^.

Adenoid tissue or tongue mass may reduce the air space and cause postural adaptations at the level of the oropharynx. A hyoid bone drop in relation to the mandible would represent an attempt to assure a relatively constant air-space diameter in the antero-posterior direction. This neuromuscular recruiting could cause changes in mandibular rest position and neck extension, thus influencing the craniofacial growth pattern^11^.

Thus, air space pattern and stability would represent major factors responsible for hyoid bone position^12^.

Since malocclusion may be caused by inadequate oral habit, such as atypical swallowing and oral breathing - hyoid bone position could serve as an important diagnostic guide^9^.

Numerous authors have studied the hyoid bone morphology and function^13^,^14^, and others have investigated hyoid bone position in relation to the cranium and the cervical spine by means of cephalometric techniques^8^,^15^, ^16^, ^17^, ^18^, ^19^, ^20^, ^21^, ^22^, ^23^, ^24^, ^25^, ^26^, ^27^, ^28^, ^29^, ^30^.

Since the studies regarding hyoid bone position represent an open field in sciences, the present study aimed at investigating the relationship between hyoid bone, mandible, cranium and cervical spine, by cephalometric means, trying to establish a relationship between hyoid bone position in oral breathing and in nasal breathing patients in order to aid in the diagnosis of alterations in the craniofacial complex, of multidisciplinary character, involving otorhinolaryngologists, orthodontists, functional orthopedists, speech therapists and physical therapists.

## MATERIALS AND METHODS

The present investigation only started after it was approved by the Research in Human Beings Ethics Committee (CEP) - FOP-UNICAMP, according to the documentation required by Resolution 196/96 of the National Committee of Research Ethics (CONEP) - National Health Council - Ministry of Health.

For this study, we used side view teleradiographies from 53 Caucasian, female individuals, selected from a pool of 450 duly enrolled children in the Municipal School Network at the city of Limeira, ranging between 9 and 12 years.

We requested authorization from the schools and also the written consent from the parents of the children who participated in this research project.

The children were evaluated by a dentist by means of an anamnesis and standard dental examination from UNICAMP. After that, the children were referred to take a lateral view teleradiography and on the same day underwent a nasofibroscopic exam. After diagnosing the predominant respiratory pattern, issued by the otorhinolaryngologist, the children were divided into control group - predominantly nasal breathers (n = 28), and experimental group - predominantly oral breathers (n = 25). Later on we carried out the cephalometric analysis.

The following criteria were used in order to select the sample from the present group: females, Class I Angle malocclusion and mixed dentition, no orthodontic treatment and/or functional orthopedic treatment of the maxillas, no extensive carious lesions, enough contrast and sharpness for a good visualization and identification of the structures that make up the tegumentary tissue, bone structures, dental elements and the hyoid bone, and no radiographic distortions.

The teleradiographies were taken laterally and in the natural head position (NHP)^31^,^32^ always by the same technician - person responsible for the Department of Dental Documentation, following the standards established by the School of Dentistry of Piracicaba/UNICAMP.

All the individuals were evaluated by the otorhinolaryngologist in charge of diagnosing the respiratory pattern. He examined the patients' ears nose and throat and did a nasofibroscopy, evaluated the questionnaire answered by the parents, the history filled out by the dentist, as well as the lateral view teleradiography, making up the standard respiratory process and classifying them as predominantly nasal (clinically normal) or predominantly oral breathers. The following material were employed for nasofibroscopy: Samsung 14” monitor; Sony 4 head VCR; Welch Allyn light source; micro camera for a Toshiba endoscope; the endoscopes used were from two models, with 0° and 30° visual angle; Pentax flexible nasofibroscope; Sony videotape; paper towel, gauze, 2% glutaraldehyde; 70% alcohol and nasal decongestant; 45% lidocaine and 10% spray xylocaine. The otorhinolaryngologist used the Ianni Filho^33^ and Wang et al.^34^ protocols in order to present the nasofibroscopic results and the exams were recorded in videotape, which became part of the Department of Pediatric Dentistry's Library at the FOP/UNICAMP.

**Figure 1:**
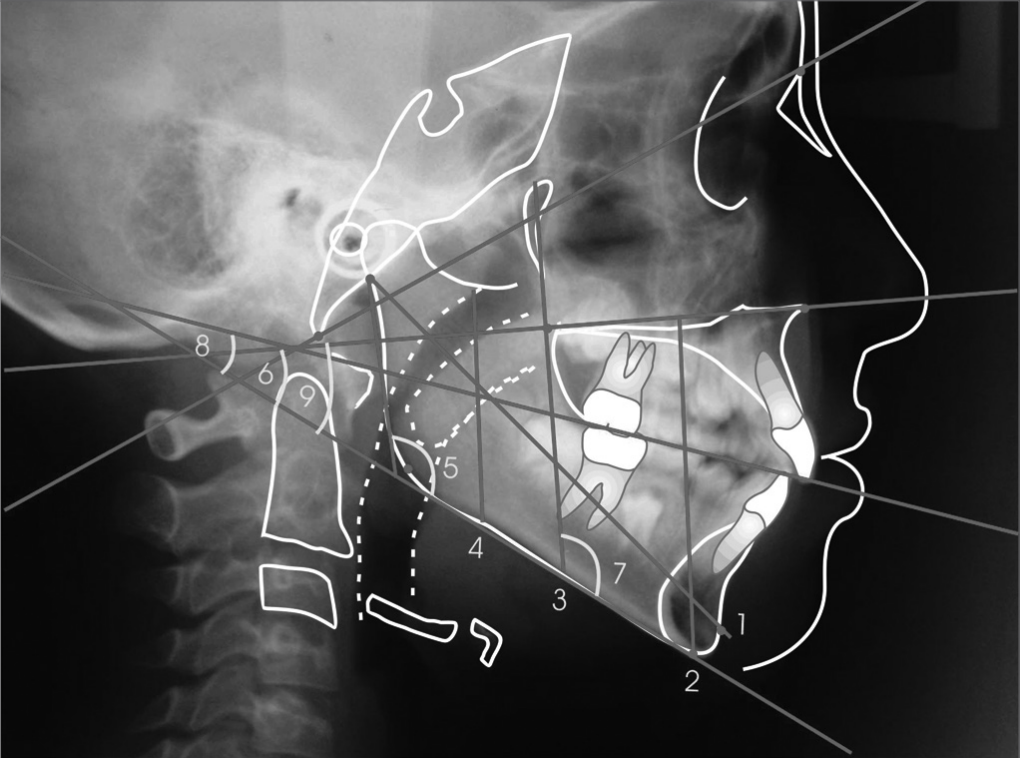
Cephalometric measures to determine mandibular position.

**Figure 2:**
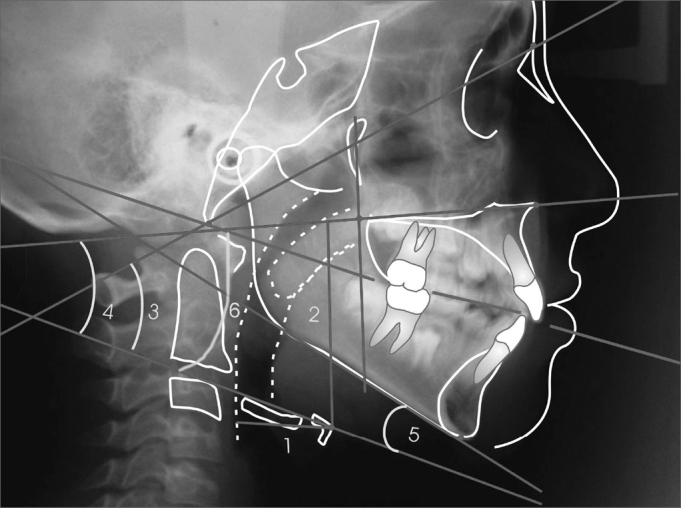
Cephalometric measures to determine the hyoid bone position.

**Figure 3:**
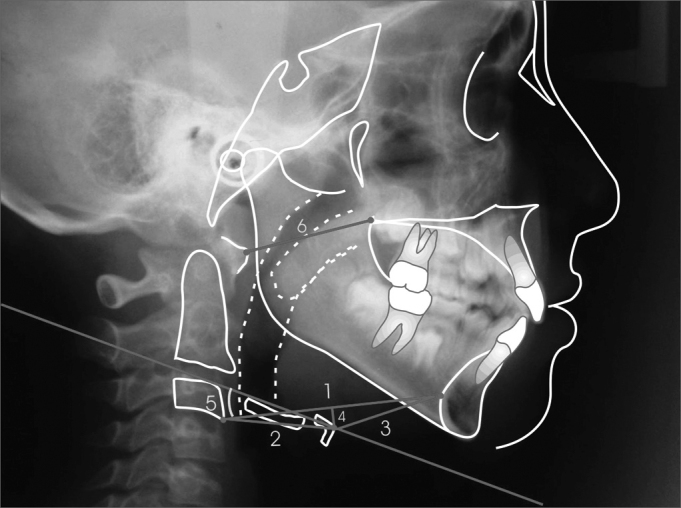
Hyoid Triangle linear and angular cephalometric measures.

The hyoid bone was cephalometrically evaluated, using craniofacial and hyoid measures^21^, adapted to the present study, and also the measures from the Hyoid Triangle^8^ (Figures 1, 2,3).

In order to evaluate the reliability of the cephalometric measures, the curves were done twice by one single researcher in one week interval, keeping the same environmental conditions and work instruments. We used the average of the values collected in the two curves. Thirty days after the curves were made, ten teleradiographies were randomly selected from individuals enrolled in this research, aiming at checking the error made between the two moments by means of calculating the error as proposed by Dalberg^35^ and advocated by Houston^36^. For data analysis, initially we obtained a descriptive analysis (mean and standard deviation), and later we applied the F test and the t Student test, with 5% significance level. The correlation between variables AA-ENP (antero-posterior distance between the first cervical vertebra and the posterior nasal spine) and C3-H (distance between the most anterior-inferior point of the third cervical vertebra and the hyoid bone body), analyzed by the Pearson^37^ correlation coefficient (1909), mentioned by Stepovich (1965)19, with a significance level of α=0.05.

## RESULTS

Calculating the error we noticed that there was no statistical significance among the moments assessed, showing reliability in the curves and measures.

Mandibular cephalometric measures' mean values and standard deviations are presented on Table 1. Average differences, as shown by the results from the “t” tests are presented on the same table, and are not significant (p >0.05).

**Table 1:** Mean value, Standard deviation and t test of the measures related to mandible position (values in degrees and millimeters).

Measures	Nasal	Oral	p*		
	Mean	SD	Mean	SD	
Ar-Pog	101.96	5.69	102.06	5.80	0.95 (NS)
PP-Me	59.59	3.43	59.85	3.90	0.80 (NS)
ENP-PM	42.87	4.45	43.65	3.48	0.49 (NS)
S-PM	44.23	3.05	43.17	2.97	0.21 (NS)
Ang. Gon.	125.16	4.34	123.40	5.04	0.18 (NS)
BaN.PM	53.41	3.51	52.32	5.10	0.37 (NS)
PTM.PM	117.18	3.39	117.80	4.64	0.59 (NS)
PP.PM	27.83	3.50	28.11	4.52	0.81 (NS)
PO.PM	15.08	3.32	14.42	2.74	0.43 (NS)

SD=Standard Deviation

*Student t test

(NS) not significant

Average and standard deviation of the measures that characterize the hyoid bone position are presented on Table 2. According to the results from the t tests presented on the same table, the mean cephalometric values from predominantly oral and nasal breathers are not statistically significant (p >0.05).

**Table 2:** Mean, Standard deviation and t test of the measures related to hyoid bone position (values in degrees and millimeters).

Measures	Nasal	Oral	p*		
	Mean	SD	Mean	SD	
d horiz. H	27.58	2.59	27.83	3.49	0.78 (NS)
d vert. H	52.41	5.45	52.95	6.46	0.74 (NS)
PH. BaN	54.30	7.42	51.44	11.33	0.29 (NS)
PH.PP	28.58	7.29	26.57	10.25	0.61 (NS)
PH.PM	1.03	6.58	-1.44	10.71	0.32 (NS)
PH.PO	16.07	7.32	12.93	10.50	0.22 (NS)

SD=Standard Deviation

*Student t test

(NS) not significant

In the present investigation we also studied cranial values that measure the hyoid bone position according to Bibby & Preston8. The data obtained from the 53 patients enrolled in the present investigation and divided in two groups are depicted on Table 3, not presenting statistically significant difference between the groups (p>0.05).

**Table 3:** Mean, Standard deviation and t test of the measures related to the Hyoid triangle (values in degrees and millimeters).

Measures	Nasal	Oral	p*		
	Mean	SD	Mean	SD	
C3-RGn	64.90	6.68	67.09	6.90	0.55 (NS)
C3-H	31.99	2.99	32.45	2.54	0.55 (NS)
H-RGn	33.70	5.09	35.53	5.76	0.23 (NS)
H-H'	1.53	5.18	2.36	5.12	0.56 (NS)
Ang. PH	22.74	7.94	20.72	12.25	0.49 (NS)
AA-ENP	32.87	3.34	32.86	2.18	0.98 (NS)

SD=Standard Deviation

(NS) not significant

* Student t test

**Figure 4:**
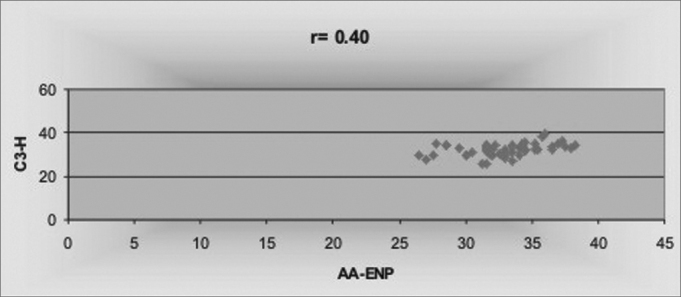
Correlation between AA-ENP and C3-H measures.

A significant, though weak, correlation (r=0.40), between AA-ENP and C3-H was found (Figure 4) representing the upper and lower limits of the air space, respectively.

## DISCUSSION

The sample was standardized in relation to gender, since it is necessary to consider sexual differences in skeletal morphology, and the basic size difference happens after puberty, with males growing more and for longer than women^38^ and most specifically in regards of cervical spine growth^15^,^17^ and hyoid bone position in relation to the mandible^30^.

In relation to the age range, mean values were of 10 years for predominantly nasal and oral breathers, we considered the high frequency with which craniofacial alterations justify the search for orthodontic treatment. Meredith1 commented that at nine years of age, 95% of the craniofacial growth has already happened. This shows the importance of the prevention aspect in the diagnostic process.

Stressing the importance of the multi and interdisciplinary approach among health care professionals involved in the diagnosis and treatment of oral breathers, we chose the nasofibroscopy carried out by the ENT, together with the teleradiography taken at a lateral view, dental assessment carried out by the investigator, interview and questionnaires answered by the parents, making up the diagnostic process of the respiratory pattern.

In a general way, the hyoid bone cephalometric measures do not show significant differences among the groups with predominantly nasal and oral breathing. This statement is in agreement with studies from Subtelny & Sakuda^18^, Bibby & Preston8, Bibby9, Kumar et al.^25^ and Kawashima et al.^29^, in which the hyoid bone presented a permanent rest position, unrelated with the respiratory pattern. Nonetheless, Adamidis & Spyropoulos^21^ found statistically significant differences in tongue, mandible and hyoid bone position among nasal and oral breathers. According to findings from the author who included oral breathers among Class I malocclusion children when compared to a control group with ideal occlusion, without evidences of nasopharynx obstruction, the respiratory pattern impacted mandible and hyoid bone positions. Authors such as Behlfelt et al.^22^,^23^, Shintani et al.^26^, Finkelstein et al.^27^ found differences in hyoid bone vertical position.

In regards of the Hyoid Triangle measures, horizontal linear measures C3-H, C3-Rgn and H-Rgn did not show significant differences between oral and nasal breathers, respectively, in accordance with Bibby^9^ and Kawashima et al.^29^. Nonetheless, qualitatively speaking, when we compared the mean values, we noticed that the C3-Rgn antero-posterior dimension (67.09mm and 64.90mm) was greater for the group of oral breathers when compared to nasal breathers. The antero-posterior dimension between the first cervical vertebra (AA) and the PNS (posterior nasal spine) was constant for both groups, since it is established in initial childhood stages^15^. Mean value was of 32.87mm and standard deviation of 3.34 for predominantly nasal breathers; and of 32.86mm and standard deviation of 2.18 for predominantly oral breathers. These results corroborate the ones from Bibby & Preston8, Ferraz et al.30, with mean values of 32.91mm, 32.83mm and standard deviation of 3.66 and 2.69, respectively. The correlation coefficient between these two horizontal measures AA-ENP and C3-H was significant and positive (r=0.40); however, less so when compared to the results from Bibby & Preston^9^ (r=0.98) and Ferraz et al.30 (r=0.56) who defined the hyoid bone as the pharynx anterior bony limit at a lower level than the PNS.

Considering mean values, it has been noticed that the hyoid bone vertical behavior in relation to C3-RGn was positioned more caudally in predominantly oral breathers, with mean value of 2.36mm and standard deviation of 5.12 when compared to nasal breathers, whose mean values was of 1.53mm and standard deviation of 5.18. This lower position, under the qualitative view point, could be interpreted as a postural adaptation at the level of the oropharynx, in an attempt to keep constant the antero-posterior diameter^11^,^12^,^22^.

Finally, the need to keep the correct ratios in the airways is one of the key factors that control hyoid bone position in individuals with a different respiratory pattern. The craniofacial complex tries to achieve a more favorable position in order to carry out its function and, therefore, adapts itself, according to its possibilities, aiming at what is easier in order to breathe properly.

## CONCLUSION

Considering the characteristics of the sample used, the methodology employed and the rigorous data analysis, it was possible to conclude that respiratory pattern did not interfere in mandibular position, or in hyoid bone position, which was maintained stable, most likely in order to assure the correct proportions of air space.

In the present investigation, quantitative studies were necessary in order to investigate changes in hyoid bone position, considering possible clinical implications of neuromuscular adaptations of oral breathing vis-à-vis the cervical spine and body posture.
